# Importance of Preoperative Vertical Geometric Analysis in Surgery for Anterior Communicating Artery Aneurysm

**DOI:** 10.7759/cureus.41322

**Published:** 2023-07-03

**Authors:** Gökhan Gürkan, Murat Atar

**Affiliations:** 1 Department of Neurosurgery, Izmir Katip Çelebi University Atatürk Training and Research Hospital, Izmir, TUR; 2 Department of Neurosurgery, Sultan Abdülhamid Han Training and Research Hospital, Istanbul, TUR

**Keywords:** skull base surgery, gyrus rectus, frankfurt horizontal plane, cerebral aneurysm, anterior communicating artery

## Abstract

Objective: In this study, we analyzed the vertical geometry of the anterior communicating artery (AComA) complex with the aim to improve the surgical site orientation during aneurysm operations. Further, the geometric data that can contribute to the success of the surgical technique applied for AComA aneurysms are reported.

Methods: Computed tomography angiography data of the cerebral arteries of 100 patients who visited our clinic were analyzed. A three-dimensional examination was performed according to the Frankfurt horizontal plane (FHP) using the RadiAnt DICOM Viewer (Medixant, Poznan, Poland), and vertical measurements of the AComA complex were calculated.

Results: The Willis polygon values were found to be consistent with those in the literature. The mean height of the AComA complex was 30.58 ± 4.80 mm according to the FHP.

Conclusions: Preoperative evaluation of the vertical geometry of the AComA complex is essential for AComA aneurysm surgery. The height of the AComA complex is a key parameter affecting intraoperative surgical site visibility. Preoperative calculation of the height of the AComA complex relative to FHP can facilitate intraoperative surgical site orientation.

## Introduction

The anterior communicating artery (AComA) is a short artery that connects the bilateral anterior cerebral arteries (ACAs). The AComA is located above the optic chiasm and corresponds to the lower part of the cistern of the lamina terminalis [1].

Based on its morphology, Yasargil classified AComA as a simple as well as a complex structure. In the simple classification, the bilateral ACA is connected to the A1 segment [2].

In the study by Rhoton, the mean AComA length was 2-3 mm, the mean AComA diameter was 1.2 mm, the mean distance from the posterior midpoint to the lamina terminalis was 11.8 ± 3.4 mm, and the mean distance from the superior surface of the optic chiasm was 2.1 ± 1.9 mm. They observed a direct correlation of the difference between the size of the right A1 and left A1 with the AComA. In other words, the size of the AComA increases with an increase in the difference between the diameters of the bilateral A1s. Therefore, a large AComA complex is usually associated with a significant difference between the diameter of the right and left A1. Further, the A1 segment in the Willis polygon has the highest incidence of hypoplasia. Moreover, A1 segment hypoplasia is strongly associated with AComA aneurysms, with a reported rate of 85% [3].

Natural head position (NHP) is a standardized and reproducible position of the head in an upright posture, the eyes focused on a point in the distance at eye level, which implies that the visual axis is horizontal. However, NHP can vary from person to person. For this reason, Frankfort horizontal plane (FHP) was developed and started to be used. FHP is defined as the horizontal plane passing through the lower edge of the orbit and the upper edge of the external auditory meatus [4].

This study aimed to contribute to the literature by analyzing the vertical geometry of the AComA complex in relation to the FHP.

## Materials and methods

**Figure 1:**
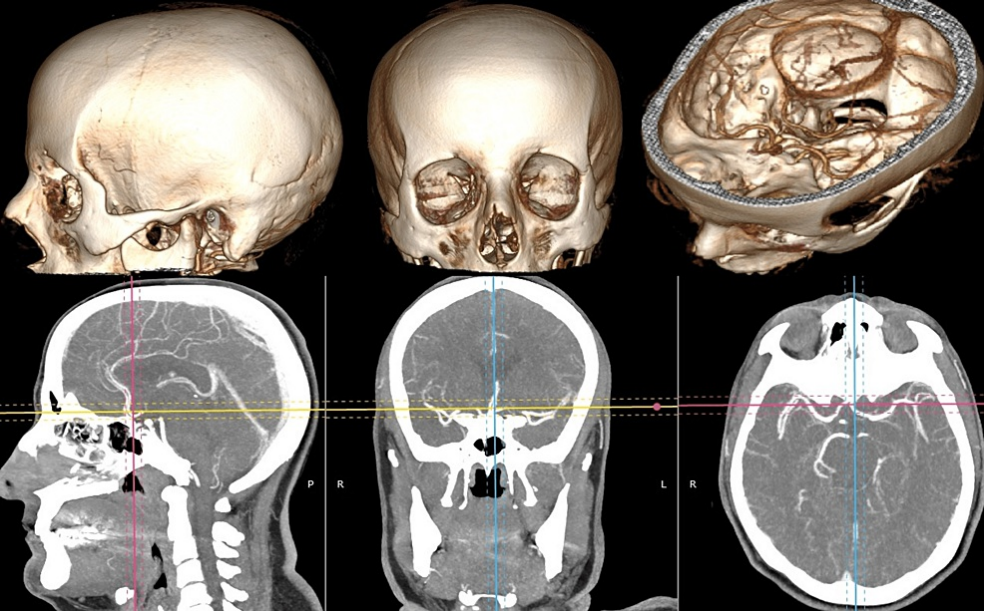
Demonstration of three-dimensional synchronized measurements according to Frankfurt horizontal plane

This study was conducted in the laboratory of the Department of Neurosurgery and Neuroanatomy, Sultan Abdülhamid Han Training and Research Hospital, Istanbul, Türkiye. We examined the computed tomography angiography (CTA) scans of the cerebral artery of 100 patients who were admitted to our hospital between October 1, 2017, and November 30, 2022. The study was approved by the Izmir Katip Celebi University Non-Invasive Clinical Research Ethics Committee, Türkiye (approval number: 0601). A three-dimensional (3D) examination was performed according to FHP using the RadiAnt DICOM Viewer (Medixant, Poznan, Poland) program and vertical measurements of the AComA complex were calculated (Figure 1).

Patients with vascular (aneurysm-arteriovenous malformation) or brain tumor pathology, those with a history of brain surgery, and those with major variations in the Willis polygon were excluded from the study. Aplasia and fetal circulation were considered major variations, whereas arterial hypoplasia, asymmetry, and arterial branching, as well as the shape differences in the A1-AComA-A2 complex were considered minor variations.

**Figure 2:**
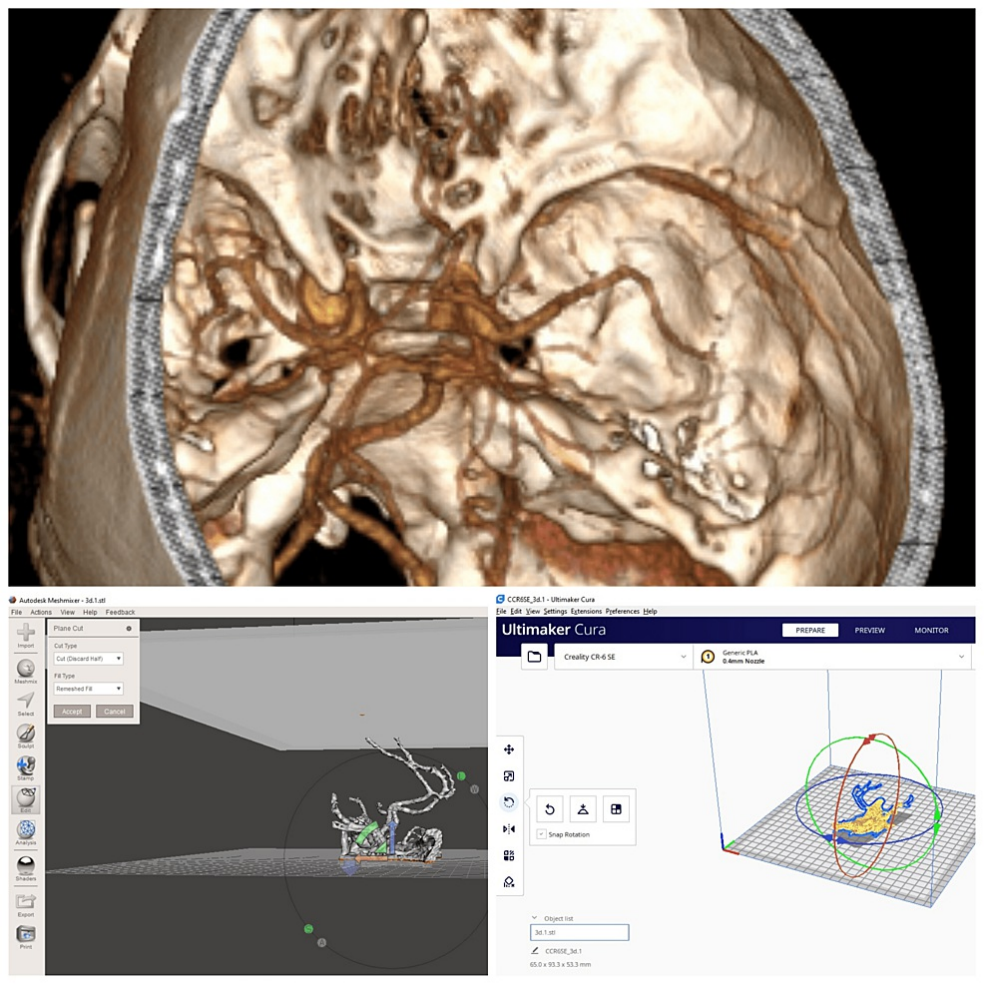
Demonstration of computer programs used for three-dimensional modeling Autodesk Meshmixer (San Francisco, California, United States); UltiMaker Cura (Ultimaker, Utrecht, Netherlands)

To emphasize the importance of measuring the height of the AComA complex relative to FHP and demonstrate the surgical orientation, 3D models of the skull and Willis polygon arterial structure of two patients each with the highest and lowest AComA height were developed. Thin-section CT and artery CTA data of the patients were prepared for 3D printing using two computer programs, the details of which are given below (Figure 2).

3D model creation

The CT data were obtained in Digital Images and Communications in Medicine (DICOM) format, enabling the formation of an interface between devices and facilitating the creation of a solid model. The RadiAnt DICOM Viewer application was used to differentiate between the skull base and vascular neuroanatomy. Skull base and vascular structures data were converted into a 3D model and exported as “.stl” files. 3D STL models obtained from RadiAnt DICOM Viewer were meshed again using the Meshmixer ™ (Autodesk, San Francisco, California, United States) software. The models prepared for 3D printing were exported and sliced using Cura software (Ultimaker, Utrecht, The Netherlands); subsequently, the Gcodes were created [5].

**Figure 3:**
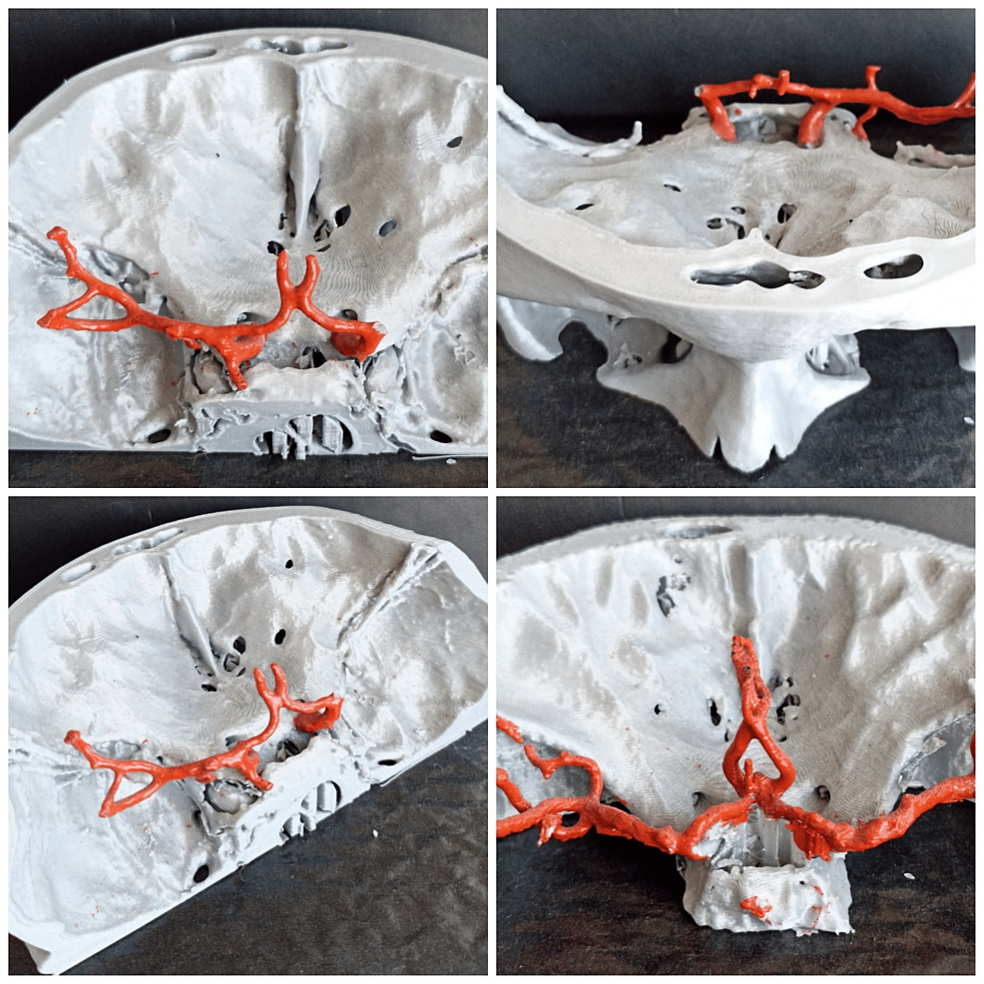
Demonstration of three-dimensional models

Polylactic acid (PLA) filament of 2.85 mm was used during the printing process following the manufacturer’s recommendations. The process settings were standardized as follows: extruder temperature, 215°C; room temperature, 24°C; primary layer height, 0.1 mm; filling rate, 20%. The 3D models were printed on a 1:1 scale [6]. They were prepared in a single session using thin section (0.3 mm) cranial CTA data and then the vascular structures were colored using special model paints. A professional photography studio was created such that high-quality 3D images of each 3D model were obtained by combining multiple focus images using Canon EOS 2000D (Canon Inc., Ota City, Tokyo, Japan) (Figure 3).

Statistical analysis

IBM SPSS Statistics for Windows, Version 22.0 (Released 2013; IBM Corp, Armonk, New York, United States) was used for statistical analysis. The mean, median, and standard deviation values for the various parameters were obtained.

## Results

Our study included 100 patients (female-to-male ratio 1:1; mean age 50.5 ± 15.4 years). The mean AComA height relative to FHP was 30.58 ± 4.80 mm. The mean AComA length and diameter were 1.54 ± 0.54 mm and 1.28 ± 0.41 mm, respectively. The mean length of right A1 was 13.55 ± 2.68 mm and that of left A1 was 12.52 ± 2.35 mm. The mean diameters of right A1 and left A1 were 1.89 ± 0.47 mm and 1.74 ± 0.34 mm, respectively. The mean length of A2 was examined in the study. The mean diameter of right A2 was 1.86 ± 0.24 mm, whereas that of left A2 was 1.77 ± 0.38 mm. Regarding the internal carotid artery (ICA) measurements, the mean diameter of right ICA and left ICA were 3.01 ± 0.56 mm and 3.13 ± 0.54 mm, respectively (Table 1).

**Table 1:** Height value of anterior communicating artery complex according to Frankfurt horizontal plane and geometric measurements of Willis polygon anterior circulation

Variables	Patients (n=100), mean±SD
Age	50,55 ± 15,42
AcomA Height (mm)	30,58 ± 4,80
AcomA Length (mm)	1,54 ± 0,54
AcomA Diameter (mm)	1,28 ± 0,41
R-A1 Length (mm)	13,55 ± 2,68
L-A1 Length (mm)	12,52 ± 2,35
R-A1 Diameter (mm)	1,89 ± 0,47
L-A1 Diameter (mm)	1,74 ± 0,34
R-Carotis Diameter (mm)	3,01 ± 0,56
L- Carotis Diameter (mm)	3,13 ± 0,54
R-A2 Diameter (mm)	1,86 ± 0,24
L- A2 Diameter (mm)	1,77 ± 0,38

After obtaining radiological measurements, 3D modeling was conducted in a neuroanatomy laboratory. Further, 3D models of the skull and vascular structures were created using CT and CTA data of two patients with the lowest and highest value of the height of the AComA complex in our cohort. These models helped determine the importance of the AComA height.

## Discussion

The Willis polygon can be divided into two sections. The AComA and A1 segments form the anterior half, whereas the posterior communicating artery (PComA) and P1 segments form the posterior half [7,8]. In a study on 100 Italian cadavers (age range, 17-84 years) by Orlandi et al., no differences were observed between males and females; however, the left arteries were longer than the right arteries [9]. In our study, the right A1 was longer than the left A1; however, this difference was not statistically significant.

In the literature, normal polygon values are as follows: A1 diameter, mean 2.6 mm (range: 0.9-4.0 mm); A1 length, mean 12.7 mm (range: 7.2-18.0 mm); AComA diameter, mean 1.5 mm (range: 0.2-3.4 mm); AComA length, mean 2.6 mm (range: 0.3-7.0 mm); and ICA diameter, mean 4.3 mm (range: 2.5-7.0 mm) [10]. The polygon values in our study were consistent with those in the literature.

In a bilateral study by Kamath et al., only the right A1 was found to be significantly longer than the left A1 [11]. In our study, the right A1 was also longer than the left A1. The larger the difference in the diameters of the bilateral A1 segments, the more likely is the development of an AComA aneurysm. Studies that compared bilateral A1 segments have found that an increase in the difference in bilateral thickness was associated with the development of AComA aneurysm. They also reported that the aneurysm was located at the junction of A1 and AComA, which was thick [12]. Patients with vascular pathology were not included in our study. The differences in the bilateral A1 measurements in our cohort were not statistically significant.

In general, approximately 40% of all AComA have a single body with a length of 1.5-8.8 mm (mean length, 4.0 mm) and a diameter of 0.2-2.5 mm (mean diameter, 1.7 mm) [1,2]. In our study, the length and the diameter of AComA were 1.54 ± 0.54 mm and 1.28 ± 0.41 mm, respectively. The AComA complexes in our cohort were morphologically simple and were a single body.

The vertical geometry of the AComA complex is not well characterized in contemporary literature. In the study by Rhoton, the optic chiasm was used as the reference point for measurements; however, obtaining this measurement from the CT and CTA data is technically challenging [3].

The only study using measurements according to FHP was conducted in 1988 by Sturz as part of their thesis for the Department of Anatomy, University of Würzburg [13]. In the cadaveric studies by Sturz, the average length of the AComA complex relative to FHP was 30.8 (21-40) mm. In our study, the height of the AComA complex relative to FHP was 30.58 ± 4.80 mm in terms of radiologic measurements. In aneurysm surgery, the height of the AComA complex is an important factor in preoperative planning. Preoperative radiologic measurements should be obtained in cases requiring gyrus rectus (GR) resection and surgical orientation.

In their study on 100 cadavers, Karatas et al. reported the following measurements: right and left A1 diameter, 1.87 ± 0.48 (0.30-3.40) mm vs. 1.96 ± 0.49 (1.00-3.20) mm; right and left A1 length, 14.44 ± 2.32 (8.10-20.00) mm vs. 13.72 ± 2.12 (8.30-19.50) mm; and the right and left ICA diameter, 3.55 ± 0.65 (2.30-5.80) mm vs. 3.45 ± 0.64 (2.00-5.00) mm [14]. Karatas et al. performed 100 CTAs and reported the following measurements: right and left A1 diameter, 2.15 ± 0.63 mm vs. 2.26 ± 0.61 mm; the right and left A1 length, 15.61 ± 2.79 mm vs. 15.13 ± 2.54 mm; AComA diameter, 1.39 ± 0.83 mm; AComA length, 1.48 ± 1.45 mm [15]. In another study of 384 patients who underwent cranial magnetic resonance angiography, the following measurements were reported: right and left ICA diameters, 4.24 and 4.32 mm; right and left A1 diameters, 1.58 mm and 1.64 mm [16]. The measurements in our study were consistent with those in the literature.

Traditionally, the GR is considered a nonfunctional gyrus. Although some studies have reported that GR resection may result in mild language and memory dysfunction, it is still considered a safe and accepted technique [17-19]. Valli et al. were the first to quantitatively measure the increased visual distance after GR resection. They reported a mean resected GR span of 7 ± 3.9 mm. Further, they showed that after GR resection, the exposed span of the ipsilateral A2 increased from 2 ± 0.7 mm to 4 ± 1.1 mm, whereas the exposed span of the contralateral A2 increased from 3 ± 1.5 mm to 4 ± 1.1 mm [20]. No such study has been done on the GR before. The literature does not provide sufficient data to determine the surgical approach in AComA aneurysm surgeries and more studies on GR are needed.

In the present study, we attempted to emphasize the importance of the relationship between the AComA complex and GR by measuring its height relative to the FHP. Excess height of the AComA complex is liable to impact surgical exploration and visual access. Finally, if exploration will be difficult, GR resection will be required. The limitations of our study are that the measurements were made in a limited number of patients and that patients with major vascular pathology were not included in the study.

## Conclusions

In this study, we emphasized the importance of measuring the height of the AComA complex. Our results indicate that preoperative evaluation of the vertical geometry of the AComA complex before surgery for AComA aneurysms may be clinically relevant for surgical planning. The height of the AComA complex relative to FHP can be preoperatively calculated as it may play a crucial role in providing or reducing intraoperative visibility. We believe this measurement may help improve surgical outcomes through careful surgical planning tailored to each patient's anatomy.
